# Epidemiology and tumor microenvironment of ocular surface and orbital tumors on growth and malignant transformation

**DOI:** 10.3389/fonc.2024.1388156

**Published:** 2024-10-03

**Authors:** Shangkun Ou, Yuan Lin, Yujie Zhang, Ke Shi, Huping Wu

**Affiliations:** ^1^ Xiamen Eye Center and Eye Institute of Xiamen University, School of Medicine, Xiamen, China; ^2^ Xiamen Clinical Research Center for Eye Diseases, Xiamen, Fujian, China; ^3^ Xiamen Key Laboratory of Ophthalmology, Xiamen, Fujian, China; ^4^ Fujian Key Laboratory of Corneal and Ocular Surface Diseases, Xiamen, Fujian, China; ^5^ Xiamen Key Laboratory of Corneal and Ocular Surface Diseases, Xiamen, Fujian, China; ^6^ Translational Medicine Institute of Xiamen Eye Center of Xiamen University, Xiamen, Fujian, China

**Keywords:** ocular surface tumor, orbital tumor, tumor microenvironment, signal pathway, MALT, mucosa associated lymphoid tissue

## Abstract

The ocular surface and orbit constitute unique microenvironments in the human body. Current advances in molecular research have deepened our understanding of tumor development in these regions. Tumors exhibit greater heterogeneity compared to normal tissues, as revealed by pathological and histological examinations. The tumor microenvironment (TME) plays a crucial role in the proliferation and progression of cancer cells. Factors from the external environment or the body’s own inflammation and microcirculation interact within the TME, maintaining a delicate balance. Disruption of this balance, through uncontrolled signal pathway activation, can transform normal or benign tissues into malignant ones. In recent years, various systemic immunotherapies have been developed for cancer treatment. This study reviews the epidemiology of ocular surface and orbital tumors include squamous cell carcinoma, basal cell carcinoma, sebaceous carcinoma and lymphoma in conjunction with their occurrence, growth, and underlying mechanisms. We propose that by examining clinical histopathological images, we can identify specific and shared microscopic features of tumors. By collecting, classifying, and analyzing data from these clinical histopathological images, we can pinpoint independent diagnostic factors characteristic of tumors. We hope this study provides a basis for future exploration of the mechanisms underlying different ocular diseases.

## 1 Introduction

Ocular surface is direct contact with the external environment, often invaded by various harmful pathogens. Ocular surface under the influence of a variety of factors in a delicate balance, but when the special events affecting environmental changes, the balance will be broken, lead to abnormal tissue cell hyperplasia or transformation to malignant tumor (1). The occurrence and development of ocular tumors can be influenced by various factors such as region, race, gender, age, and epidemiology (2–5). In addition, the structure and function of the orbit and ocular surface are closely related, with the lacrimal gland being a common site for orbital tumors (6). Lacrimal gland lesions form a subset of orbital tumors and can exhibit benign or malignant characteristics (7). Local and systemic immunological factors can manifest in lacrimal gland tumors (8). A study reported that primary lacrimal gland lymphoma is confined to the orbit and can occur without any systemic findings (9).

During a 6-year study, 9,633 new patients were diagnosed with eye tumors, representing 0.8% of all cases. Tumors were classified as benign in 65% of cases, pre-malignant in 3%, and malignant in 32%. The most common tumors identified were retinoblastoma, ocular surface squamous neoplasia, and conjunctival nevus. Age-specific analysis revealed that retinoblastoma was the most common tumor in children aged 0-10 years, conjunctival nevus predominated in the 11-30 years age group, and ocular surface squamous neoplasia was most common in patients over 30 (10). Furthermore, an analysis of primary orbital tumors identified 1,000 cases, with 72% being benign and 28% malignant. Histopathological confirmation was obtained for 55% of benign and 99% of malignant tumors. The most common benign orbital tumor was idiopathic orbital inflammation, followed by IgG4-related ophthalmic disease, cavernous venous malformation, and pleomorphic adenoma (11). Lymphoma was the most prevalent malignant tumor (70%), with adenoid cystic carcinoma (7%) and solitary fibrous tumor (5%) also being significant. Orbital lymphomas, predominantly of B-cell origin (97%), constituted 50-60% of ocular adnexal lymphomas, with extranodal marginal zone B-cell lymphoma (EMZL) being the most common subtype (59%). Gender distribution varied by lymphoma subtype, with EMZL and follicular lymphoma showing a female predominance, while mantle cell lymphoma had a strong male predominance. Prognosis and treatment decisions were guided by the histopathological subtype and clinical stage, with radiotherapy being the treatment of choice for solitary low-grade lymphomas and chemotherapy, with or without radiotherapy, preferred for disseminated and high-grade lymphomas (12).

The clinical manifestations of ocular surface tumors encompass a wide range, including various forms of epithelial, stromal, cartilaginous, and secondary tumors (13). Ocular surface squamous neoplasia, conjunctival melanoma, and conjunctival lymphoma are the most common ocular surface malignancies (14). Due to their distinct clinical features, high resolution optical coherence tomography (HR-OCT) can be used in clinics to differentiate between epithelial ocular surface tumors like ocular surface squamous neoplasia (OSSN) and subepithelial tumors such as conjunctival lymphoma and melanoma. *In vivo* confocal microscopy can complement HR-OCT by providing cellular and surface details, while ultrasound biomicroscopy assesses tumor depth and thickness in larger, highly pigmented lesions and detects intraocular invasion (15). Chemotherapeutic agents such as 5-Fluorouracil (5-FU), mitomycin-C (MMC), and interferon (IFN)-alpha2b are currently used in clinical practice for the topical treatment of ocular surface tumors, particularly OSSN (16). Studies have shown that non-invasive examinations can monitor tumor treatment progress and assess the timing for surgical intervention (17, 18).

Chronic inflammation is a predisposing factor for metaplastic changes and ultimately dysplasia. OSSN may arise in chronic inflammation on the ocular surface (19). In addition, vascular endothelial growth factor is associated with reduced immune response and impaired anti-tumor activity (20). Through immunohistochemistry, certain proteins can be significantly overexpressed in both invasive and non-invasive lesions. By comparing poorly differentiated and moderately differentiated tissues, researchers can determine whether the target protein impacts tumor development and progression (21). Genetic factors may interact with environmental risk factors in developing multifactorial ocular surface diseases (OSDs) such as autoimmune diseases, allergies, tumors, and dry eye disease. Environmental or genetic susceptibility genes and dysregulation influence the occurrence and progression of ocular surface and orbital tumors. Approaches to monogenic OSDs based on genes and multifactorial OSDs with genetic susceptibility, such as immune-mediated diseases and tumors with known or potential genetic risk factors, are critical for understanding these processes (22).Pathological histological examination is the gold standard and can differentiate other proliferative comorbidities (23). Pathway validation based on pathological examination is widely used in exploring the mechanisms of ocular surface and orbital tumors. Studies have shown that, in addition to mutations in the mitogen-activated protein kinase (MAPK) pathway, multiple driver mutations have been detected in various tissue cell tumors. These low-grade or high-grade mutations indicate a potential high risk of malignant transformation (24).

Surgical resection with frozen section examination for margin control is the primary treatment for ocular surface and orbital carcinomas. Exenteration may be needed for orbital invasion (25). High-risk patients might require adjuvant radiotherapy, and sentinel lymph node biopsy can be considered for those at risk of lymph node metastasis (26). Topical chemotherapy, such as MMC, 5-Fu, or IFN-alpha-2b, is used for conjunctival primary or residual *in situ* disease (27). For metastatic or locally advanced basal or squamous cell carcinoma unamenable to surgery or radiotherapy, targeted therapies—hedgehog pathway inhibitors for basal cell carcinoma (BCC) and epidermal growth factor receptor inhibitors for squamous cell carcinoma—are effective in managing disease progression (28). Therefore, integrating clinical observations, diagnostic instruments, and molecular testing information is essential for personalized and precise treatment of ocular surface and orbital tumors. This represents a novel therapeutic approach that bridges basic research and clinical practice. In this study, we review the main mechanisms and influencing factors of various clinical manifestations by combining advances in tumor epidemiology, histopathology, and the tumor microenvironment (TME). Our review provides valuable insights for the molecular diagnosis and treatment of ocular diseases.

## 2 Epidemiology of ocular surface and orbital tumors

The clinical diagnosis of ocular surface tumors can be challenging due to their similar signs and symptoms. Therefore, collecting more cases and differentiating between pathological types and epidemiological changes is crucial to enhance the identification of these tumors. Benign tumors are frequently found in the eyelid area, with the conjunctiva, orbit, cornea, and limbus following suit in occurrence. The 8673 ocular tumors reported by Dai, J (29) et al. in 1999 and the 7910 ocular tumors and tumor-like lesions reported by Wang, L (7) et al. in 2019 obtained similar results. We aimed to elucidate the reason behind the limited diversity observed in the occurrence of eye tumor sites. The high incidence of eyelid tumors could be attributed to the intricate nature of the eyelid’s placement in the outermost layer of the eye’s structure and its constant exposure to the external environment, which may facilitate the growth of tumors.

Previous research has indicated that ocular tumors in children are linked to genetic factors and an abnormality in embryonic growth processes. Eyelid and ocular surface tumors are the most common types of ocular tumors in epidemiological studies (30). Most eyelid and ocular surface tumors are benign; a few are malignant. Intraocular and orbital tumors are mainly malignant (31). Calcified epithelioma is a common benign tumor in children’s eyelids. The cortical differentiation of the epidermis forms it and is a benign tumor in the deep skin (32). The most common malignant tumors on their eyelids and ocular surfaces in individuals aged 65 and above are squamous, basal, and melanoma. These tumors are frequently found in the eyelid and orbit (10).

Cavernous hemangioma is the most frequently occurring orbital site, typically affecting young individuals and presenting with slow proptosis as the main symptom. The histopathological imaging features of cavernous angioma involve irregular expansion of vascular spaces, partial capillary hyperplasia, and infiltration of interstitial plasma cells (33). Adult orbital malignancies primarily comprise lymphoma, a proliferative disease of immune cell tumors with multiple pathological types. Most cases are diagnosed as mucosa-associated lymphoid tissue (MALT) lymphomas, considered low-grade malignant lymphomas (34). Clinical symptoms and imaging studies can be challenging to differentiate from other conditions, such as specific inflammation, leading to misdiagnosis. Accurate diagnosis requires pathological examination and immunohistochemistry (12).

Tumors in the eyelids are among the most common ocular tumors, often caused by external contact or stimulation. Unfortunately, many of these tumors can be difficult to distinguish from inflammatory lesions, and malignant tumors can even metastasize and damage ocular health (35). Recent studies have shown that the number of patients with eyelid tumors is increasing each year, particularly for benign tumors like seborrheic keratosis and xanthoma. This could be due to changes in people’s lifestyles, cultures, and material consumption levels, as well as an increase in metabolic disorders among the population (36). For example, xanthoma, a systemic disease that often manifests as skin lesions, frequently appears on the eyelids (37). In malignant tumors, distinguishing between melanocytes can be challenging, and most proliferative lesions may develop into malignant melanoma if specific conditions are met. Basal epithelial cells may also have the ability to produce melanin, which can lead to malignancy (38). BCC is a common ocular surface malignancy occurring almost on the eyelids. Usually, nodules with pearl margins, central ulceration, and telangiectasia vessels. Risk factors include old age, sun exposure, and immunosuppression, with high recurrence rates (39). Moreover, sebaceous carcinoma (SCC) often affects older people, usually from the eyelid glands, with early symptoms of mild and insidious development (40).

Studies have shown that ocular surface microbes, temperature, and ultraviolet (UV) light can impact the conjunctival microenvironment (41). These Studies have shown that ocular surface microbes, temperature, and UV light can impact the conjunctival microenvironment (42). C.L. Shields (43) et al. investigated 5002 patients and found that identifying benign and malignant conjunctival tumors had valuable clinical features. However, experience based on clinical work still requires a histopathological diagnosis. Goblet cells and lymphatic vessels are widespread in the conjunctiva, and irritation or blockage creates cysts. Conjunctival cysts are divided into dermoid and epidermoid, which can occur after surgery due to the invagination of conjunctival epithelial cells, and both capsule walls can be coated with laminated squamous epithelium. Unlike congenital cysts, goblet cells are standard cells of the cystic wall, where, in a few cases, the wall is composed entirely of goblet cells, and the cavity is filled with jelly samples (44). Sebaceous adenocarcinoma is most commonly found in elderly patients and those with conjunctival issues. Most cases occur in the area surrounding the eye, where the sebaceous glands are most abundant (45).

The cornea plays a crucial role in the path of light, and maintaining its structure and function is essential for normal vision and refraction. The development of corneal tumors can result in significant visual impairment or even blindness. Malignant tumors in the cornea are also at risk of metastasizing (46). Distinguishing between corneal neoplasia and hyperplastic tumor-like lesions poses a clinical challenge, with dermoid tumors being the most frequent benign tumors found in the cornea and limbus (47).

The limbus is the boundary between the clear cornea and the white sclera, covering the conjunctiva. Research has shown that limbal stem cells can experience malignant degeneration like other tissues in the limbus (48); recurrent epithelial defects may be related to the limbal stem cell defect (49). Due to the limbal pathological changes’ unique anatomical structure and location, there are many misunderstood conditions, including Mooren ulcers (50) and corneal dystrophy (51).

## 3 Research progress of TME and ocular tumor properties

The TME plays a crucial role in the proliferation and progression of cancer cells. Tumor cells rely on autocrine and paracrine secretion to adapt and sustain their growth within the TME. Additionally, factors such as structural and functional changes and alterations in metabolism can impact the development of tumors by influencing the state of both local and systemic tissues (52). Environmental factors, such as UV exposure and oxidative stress, can trigger abnormal changes in ocular surface cells. Specifically, UV radiation can initiate phosphorylation events that activate critical growth factor receptors like epidermal growth factor receptor, tumor necrosis factor receptor, and interleukin 1R (53). Class III histone deacetylases and sirtuin 1 are associated with tumorigenesis and conjunctival epithelial tumor progression (54).

The TME plays a crucial role in regulating the metastatic potential of most cancers. Malignant cells and various non-malignant cells make up solid tumors, forming what is known as the “tumor stroma.” This stroma is essential to the malignant process and exhibits pro-tumorigenic and antitumorigenic behaviors (55). epithelial-mesenchymal transition (EMT) is a critical phenomenon in epithelial malignancies’ progression, invasion, and metastasis. EMT is characterized by the loss of epithelial morphology and the acquisition of a highly aggressive mesenchymal phenotype, which contributes to the invasiveness and metastasis exhibited by tumors (56).

The presence of inflammatory factors, growth factors, and proteases secreted by senescent cells as part of the aging-associated secretory phenotypes can create a microenvironment that promotes the progression of tumors. As we age, this cancer-prone condition becomes more favorable for tumor growth. Studies have shown that eyelid adenocarcinoma and BCC exhibit reduced levels of protective protein complex, shortened telomere length, overexpression of Ki-67 and B-cell lymphoma-2 (Bcl 2), and P53 mutations. This implies that these factors contribute to the development of meibomian adenocarcinoma and BCC (57). The microenvironment of embryonic S cells plays a crucial role in regulating the aging process of normal cells by controlling Phosphatidylinositol 3-kinase (PI3K) signaling in both directions. This regulation helps suppress the malignant behavior of uveal melanoma cells and controls the environment that promotes tumor-promoting senescence. The PI3K/protein kinase B (AKT) pathway is known to promote the development and progression of tumors, particularly in uveal melanoma (UVM) (58). Proteinase-activated receptors found on the surfaces of different types of tissues have been observed to regulate PI3K signaling, resulting in a diverse range of standard and disease-related functions, such as embryogenesis, inflammation, and cancer. Consequently, the microenvironment of embryonic stem (ES) cells effectively suppresses the PI3K signaling pathway, offering powerful anti-tumor effects while keeping healthy somatic cells unaffected (58). Tyrosinase-related protein 1 (TYRP 1) is a crucial marker in melanogenesis that boosts melanin production and guards against melanocyte death. As melanogenesis is induced, hypoxia-inducible transcription factor -1 α (HIF-1 α) is activated, which can influence the behavior of melanoma cells and their surroundings. Additionally, melanin functions as an oxygen scavenger, creating hypoxic conditions that further stimulate tumor growth during synthesis. Knocking out the HIF-1 α gene decreases cell invasion, migration, and adhesion in UVM cell lines. The melanogenesis pathway induces a substantial increase in hypoxia, and a correlation exists between TYRP 1, hypoxia, and the more aggressive behavior of UVM cells (59).

Cancer-associated fibroblasts (CAFs) are a specialized group of cells found within the TME that can stimulate tumor growth and angiogenesis and contribute to inflammatory responses and metastasis during tumor progression. CAFs are normal fibroblasts that have undergone significant changes due to prolonged exposure to cancer cells, which can significantly impact the outcome of the tumor. Normal fibroblasts are elongated cells typically found in connective tissue and play a vital role in the synthesis and maintenance of the extracellular matrix (ECM). These cells are crucial for maintaining normal tissue homeostasis and are involved in various biological processes, such as wound healing and aging. Conversely, CAFs are found either at the tumor margin or within the tumor itself, where they promote tumor transformation. One key factor contributing to the tumor-promoting effects of CAFs is the secretion of CXCL12 (SDF 1-a), a chemokine that can induce angiogenesis and enhance the proliferation of cancer cells (60).

There are studies describing biochemical pathways between inflammation and cancer and demonstrating that inflammation increases tumor progression and supports metastatic spread (61). Tumors have been found to affect the use of the inflammatory microenvironment by various means. Immune cells in this environment tend to promote tumor progression rather than provide a robust anti-tumor response. As a result, the activation of these immune cells causes the secretion of inflammatory mediators that produce factors that fuel the tumor within the microenvironment. Given the infiltration of immune cells within and around the tumor, the inflammatory microenvironment is believed to be a significant prognostic parameter for cancer (62).

The intricacies of tumor microcirculation remain a topic of ongoing research and are not yet entirely comprehended. Tumor angiogenesis, which is the formation of new blood vessels, is initiated by releasing chemical signals from tumor cells during their rapid growth phase. This prompts the reactive blood vessels within the neighboring tumor tissue to respond. The vasculature of neoplastic lesions displays both functional and structural disparities and varies considerably from the nearby unaffected tissue. While the normal vasculature is organized into layers, the tumor vasculature is immature, convoluted, and excessively permeable due to abnormal basement membranes and fewer myocytes and endothelial cells (63).

Some tumors are estrogenic, acquiring a resilient immune-escape trait without undergoing selective T-cell pressure. Additionally, tumor-specific T cells, such as CD4T and CD8T cells, can stimulate the development of mutated tumor cells that evade detection. These findings indicate that intratumoral T cells are undergoing active immunosuppression, which hinders the enhancement of antitumor immunity (64). When tumors grow within the anterior chamber, they may escape the immune system’s surveillance and T cell pressure. This immune escape happens due to the unique ocular environment’s influence on the genes expressed in eye-derived tumor cells (65).

When evaluating tumors within the TME, it is essential to consider the two main types of tumors: immune cells and stromal cells. In ocular tumors, the loss of brca 1-associated protein-1 (BAP 1) expression has resulted in T-cell infiltration. Additionally, Treg, CD4+, CD25+, and Forkhead box protein P3 (FoxP3)+ have been identified as critical inhibitors of Th1 and cytotoxic T lymphocyte responses, which are significant means of tumor evasion in many cancers (66). The intricate TME, consisting of tumor-infiltrating immune cells, cytokines, and chemokines, profoundly impacts tumor growth and progression. Local immunosuppressive factors play a significant role in the metastasis of eye tumors. The TME can inhibit T-cell initiation through immune mechanisms, thereby regulating tumor generation and growth. Mutation or deletion of specific genes in eye tumors may be linked to various inflammatory phenotypes, potentially leading to tumor angiogenesis.

The TME is influenced by intrinsic factors, including genetic mutations that can trigger particular immune responses. One such example is the BAP 1 tumor suppressor gene, which, when mutated, elevates the risk of metastasis in UVM. Diminished expression of BAP 1 corresponds with an immunosuppressive microenvironment and T-cell infiltration in UVM, potentially via a nearby pro-inflammatory pathway like the NF-kB pathway (67). According to the immune infiltration analysis of UVM, neutrophil infiltration is the most promising prognostic factor, with nearly all associated with this type of infiltration. The tumor-promoting effects of neutrophils are mediated through various mechanisms, including their involvement in angiogenesis through the expression of matrix metalloproteinases like MMP 9. On the other hand, neutrophils can also hinder the antitumor response of CD8+ T cells by releasing granules and arginase and expressing PD-L1 (68). In addition, the B7 family exhibits significant dysregulation in UVM, negatively correlating to methylation levels. The expression of B7 family members is linked to immune infiltration and prognosis, and they play a crucial role in the TME. Monocytes and macrophages in UVM show higher expression levels of B7 family members than other cell types. These members primarily influence the immune response and visual perception. B7 family dysregulation in UVM is indicative of poor prognosis and has an impact on the tumor immune microenvironment. Bioinformatics analysis suggests that B7-2 could work as a marker of macrophages in UVM, significantly influencing metastasis, prognosis, and immune cell infiltration. Therefore, it is likely to play a crucial role in the development and progression of UVM (69).

The current method for determining the TME of ocular tumors involves bioinformatics cell-type enrichment analysis. Research has indicated that conjunctival melanoma is characterized by an abundance of melanocytes, pericytes, and various immune cell types, such as plasmacytoid dendritic cells, natural killer T cells, B cells, and mast cells. However, the differentially expressed genes primarily relate to inhibiting apoptosis, proteolysis, and response to growth factors. POU3F3 and BIRC5 are among the top expressed genes associated with inhibiting apoptosis. Additionally, CENPK, INHA, USP33, CASP3, SNORA73B, AAR2, SNRNP48, and GPN1 have been identified as prognostic-related factors (70).

## 4 Epidemiology, cell, and molecular pathway related to the TME

The most upregulated genes and pathways in hybrid cells were associated with enhanced cell motility, cytoskeletal rearrangement, immune evasion, and altered cellular metabolism. In both tumor tissues and patient-matched peripheral blood, gene expression was validated by confirming corresponding protein expression in key pathway categories. This included thymosin, beta 10 (TMSB10) for cell motility, CD74 for immune evasion, and glutathione peroxidase 1 (GPX1) for metabolism. Notably, TMSB10 and GPX1 were significantly more expressed in disseminated hybrid cells compared to circulating tumor cells. Furthermore, CD74 and GPX1 were found at higher levels in disseminated hybrids than in tumor-resident hybrids. Hybrid cells also expressed ligand-receptor signaling pathways such as GAS6-AXL, CXCL12-CXCR4, LGALS9-P4HB, and IGF1-IGFR1, which are implicated in promoting metastasis (71).

An analysis of 114,927 cells across 16 identified cell types revealed that epithelial cells, M2 macrophages, and regulatory T cells were predominant in tumors harboring ErbB pathway mutations. Within these tumors, distinct epithelial cell subtypes were observed, with subtypes 1 and 2 predominantly found in adenocarcinoma, and subtype 4 in adenosquamous carcinoma. Tumors with ErbB mutations had larger populations of epithelial subtypes 1 and 2, which expressed higher levels of secreted midkine (MDK) than tumors without these mutations (72). Elevated MDK interacted with its receptor LRP1, expressed by tumor-infiltrating macrophages, promoting their differentiation into immunosuppressive M2 macrophages. This crosstalk induced macrophage-secreted CXCL10 to interact with its receptor CXCR3 on regulatory T cells, exacerbating immune suppression in gallbladder carcinoma (GBC) with ErbB mutations. High MDK levels were associated with poor overall survival in GBC patients (73).

In uveal melanoma, poor prognosis was linked to tumor-associated macrophages (TAMs), high mean vascular density (MVD), PAS-positive extravascular matrix patterns, and advanced patient age. These factors influenced histologic tumor growth, particularly the presence of M2 macrophages and their cytokine profiles. In a mouse model, older mice showed a higher prevalence of prognostically significant extravascular matrix patterns and M2 macrophages in untreated tumors compared to younger mice. Additionally, M2-conditioned tumors had increased TAM presence, higher collagen IV levels, and elevated MVD, indicating that an M2-dominated TME contributes to a more aggressive tumor phenotype. This suggests that while age may provide a tumor-favoring basis, the pro-angiogenic and tumor-promoting effects are more directly attributed to the M2-rich TME. The study also found that COX-2 contributes to the invasive properties of BCC, although macrophage polarization was not a major factor in aggressive behavior. Other inflammatory components, distinct from TAMs, appeared to contribute to tissue destruction and the invasive growth pattern observed in fibrosing BCC (74).

## 5 The role of different components of TME in the occurrence and metastasis of ocular surface and orbital tumors

### 5.1 Basal cell carcinoma

#### 5.1.1 The NF- κ B signaling pathway

NF- κ B is a critical player in regulating crucial cellular functions and significantly impacts tumors’ angiogenesis, inflammation, and immune response. The signaling pathway of NF- κ B is critical in both inflammation and tumorigenesis, and it is closely regulated by the I κ B-kinase (IKK) complex, which includes two catalytic subunits: IKK α and IKK β. Research has shown that IKK α can be found in the nucleus of BCC and non-malignant diseases, where it can directly bind to the promoter of inflammatory factors and the stem cell marker LGR 5. It then upregulates LGR 5 expression by activating the signal transducer and activator of transcription 3 (STAT 3) signaling pathway during cancer progression (75). NF-κB1 and p50 can act as an anti-inflammatory transcription factor by inhibiting pro-inflammatory gene expression while enhancing anti-inflammatory gene expression (62).

#### 5.1.2 Wnt/β -β-catenin signaling pathway

The Wnt signaling pathway can cause several human cancers and developmental abnormalities, as it relies on β-catenin to initiate the signaling process. This pathway has been linked to many cancers due to the abnormal activation of the canonical Wnt and β-catenin signaling pathways. This pathway has also been observed in melanoma and BCC cases, where the Wnt protein is overexpressed and β-catenin contains a stable mutation. The Wnt/β-catenin pathway promotes cell growth, morphogenesis, and stem cell maintenance. It stimulates the stemness of rare cell populations in tumor volumes, leading to cancer resistance to conventional chemotherapy. This results in disease recurrence and metastasis (76).

#### 5.1.3 JAK/STAT 3 signaling pathway

The STAT protein is crucial in programmed gene expression during various biological processes, including embryonic development, programmed cell death, organogenesis, innate and adaptive immunity, and cell growth regulation. However, when the STAT 3 signaling is constitutively activated, it can lead to tumorigenesis by inhibiting apoptosis and promoting cell proliferation, as well as inducing angiogenesis in BCC through the JAK/STAT 3 and PI3-Kinase/Akt pathways via the upregulation of secreted bFGF by IL-6 (77).

#### 5.1.4 COX-2

Based on research, COX-2 is a contributing factor to BCC invasion. While macrophage polarization does not seem to be a significant player in invasive behavior, other inflammatory components beyond tumor-associated macrophages seem to be at play in tissue destruction. For instance, fibrotic basal cells exhibit a notably more profound inflammatory response in the surrounding tissue, leading to an invasive growth pattern (74).

### 5.2 Squamous tumor

#### 5.2.1 HIF-1α

At low oxygen levels, hypoxia-inducible transcription factor (HIF) becomes activated and is responsible for maintaining oxygen balance within the body. The regulation of angiogenesis by hypoxia and HIF is crucial in the development and progression of tumors, as tumor cells often outgrow their oxygen and nutrient supply. Increased expression of HIF-1 α in ocular malignancies has been linked to unfavorable patient prognosis, defined as tumor recurrence, metastasis, or death. Studies have shown that malignant squamous cell carcinoma of the ocular adnexa exhibits significantly higher HIF-1 α expression than benign papillomas, with strong expression in tumor cells but lower in papillomas (78). On the other hand, malignant melanoma and lymphoma showed similar HIF-1 α expression to nevi and reactive lymphoid hyperplasia. This is associated with increased downstream factors of HIF-1 α and a trend toward an unfavorable clinical outcome. Additionally, many proangiogenic factors were significantly elevated in squamous cell carcinoma, including angiogenesis 4, erythropoietin, secreted frizzled-associated protein 1, and vascular endothelial growth factor (79). Among these biomolecules, vascular endothelial growth factor and erythropoietin are maturation factors for tumor angiogenesis, and these HIF-1 α-induced factors create a pro-angiogenic microenvironment in SCC, which may promote angiogenesis, tumor growth, and possibly metastatic affinity (80). While HIF-1 α signaling may make malignancy more aggressive and lead to worse clinical outcomes, it is also possible that intrinsically aggressive tumors result in lower oxygen tension, resulting in more pronounced HIF-1 α activation (81).

#### 5.2.2 Telomerase reverse transcriptase

Nearly 50% of ocular surface squamous tumors harbor mutations in the TERT promoter, and the spectrum of these mutations suggests that they are primarily induced by exposure to UV radiation. The abnormal overexpression of telomerase resulting from UV-induced TERT promoter mutations significantly contributes to the development of ocular surface squamous tumors (82).

#### 5.2.3 P16

Kobalka et al. studied nuclear and cytoplasmic p16 immunoreactivity in ocular surface and periorbital squamous tumors, with Peter J. Strong being one of the contributors. The study found that nearly all ocular surface squamous lesions showed diffuse positivity and a strong expression of p16 in both ocular surface and periorbital squamous tumors. However, further examination is needed to investigate the relationship between extensive cytoplasmic and nuclear reactive p16 expression in the ocular surface and periorbital sites and HPV infection, especially regarding histological examination of eyelid biopsy *in situ* squamous cell carcinoma (83).

#### 5.2.4 Protein phosphatase 5

PP5, a protein phosphatase, supports cell proliferation, differentiation, and development. Through histological analysis and *in vitro* co-transfection studies, it was observed that the expression of p53 was significantly increased in fibroblast growth factor 7 (FGF-7) in Tg-hPP 5 mice. Co-transfection of PP5, p53, and FGF-7 promoter-driven luciferase revealed that PP5 promoted FGF-7 expression, while p53 inhibited FGF-7 expression. This suggests that PP5 overexpression inhibited p53 phosphorylation, reducing its tumor-suppressive function and increasing the expression of FGF-7. These findings demonstrate that PP5 plays a vital role in corneal hyperplasia development, and its downregulation may be a potential therapeutic target for corneal hyperplasia and ocular surface squamous neoplasia (OSSN) therapy (84).

#### 5.2.5 P53

Hyper-methylation of lamin genes and decreased mRNA expression are promising biomarkers for detecting high-risk OSSN patients. In the development of OSSN, evidence suggests that p53-lamin-mediated signaling is involved, as there is an abnormal methylation of lamin and simultaneous mutant expression of p53. Squamous cell carcinoma cases showed a strong nuclear positivity in mutant p53 immunoexpression. The link between stratified protein methylation and mutant p53 expression indicates that p53-stratified protein-mediated signaling is also potentially involved in developing OSSN (85).

### 5.3 Sebaceous carcinoma

#### 5.3.1 The MAPK signaling pathways

Human epidermal growth factor receptor 2 (HER 2) protein is a transmembrane receptor with tyrosine kinase activity that, when activated, triggers various intracellular pathways. These pathways can inhibit apoptosis, promote cell proliferation, stimulate tumor-induced neovascularization, and activate cancer invasion and metastasis (86). A gene expression profiling study has linked dysregulation of the mitogen-activated protein kinase (MAPK) pathway with eyelid sebaceous gland carcinoma. The RAS-RAF-MAPK pathway is the primary pathway activated by HER 2, with KRAS being the most frequently mutated gene involved in this pathway. KRAS mutations play a vital role in the MAPK signaling pathway in skin cancer (87). In eyelid sebaceous carcinoma, HER 2 gene amplification and KRAS alteration are compared to other sebaceous tumors, with HER 2 protein overexpression being unique to eyelid sebaceous carcinoma. HER 2 gene amplification and HER 2 protein overexpression are standard in eyelid sebaceous carcinomas and distinguish them from other eyelid tumors (88).

#### 5.3.2 ZEB2

ZEB 2, also known as Smad interacting protein 1, interacts with the E-cadherin gene (CDH 1) to mediate its transcriptional repression. Earlier reports have indicated the loss of e-cadherin and its promoter hypermethylation status in eyelid sebaceous carcinoma (89).

#### 5.3.3 Programmed cell death-ligand 1/programmed cell death 1

It has been observed that PD-L1 and PD1 are expressed together in immune cells, and they play a crucial role in regulating autoimmunity and tolerance. The PD-L1 pathway helps to maintain a balance between T-cell functions in inflamed tissues and prevents autoimmunity (90). However, cancer cells tend to overexpress PD-L1, which then binds to the PD1 receptor on T cells, inhibiting their activation and inducing the production of cytokines like IFN γ and IL 2. This ultimately weakens the T cell response to tumor antigens and may even contribute to the aggressive nature of eyelid sebaceous carcinoma (91). The study shows a strong correlation between PD-L1 expression and metastatic and poorly differentiated sebaceous carcinoma. PD-1 and PD-L1 were also observed to highlight the immune response in both tumor and stromal cells. The study also found that the expression of PD-L1 and PD-1 in both tumor and stromal cells was associated with decreased patient survival (92). The proteins PD-1 and CTLA-4 may also work together to regulate the body’s adaptive immune response (93). CTLA-4, in particular, is a crucial immune checkpoint molecule that binds to CD80 and CD86 and is present in chronic inflammation and various cancers. This dysregulation of the immune response suggests that these proteins could serve as a valuable prognostic marker for identifying eyelid sebaceous carcinoma (64, 92).

#### 5.3.4 Tp 53/RB 1 and MYC

Identifying TP53, ZNF750, RB1, and PCDH15 mutations in ocular adnexa is likely due to loss-of-function mutations, which could have diagnostic and prognostic significance (94). Notably, most sebaceous carcinoma cases affecting the ocular adnexa exhibit MYC protein expression. Previous research has established TP53 and RB1 changes as the most prevalent genetic anomalies in sebaceous carcinoma, and there is evidence suggesting that MYC may also play a role in the development of these tumors (95).

#### 5.3.5 Vimentin

Vimentin upholds the structural integrity of normal mesenchymal cells and provides stress resistance. This protein is expressed in many cells, such as fibroblasts, endothelial cells, macrophages, neutrophils, and leukocytes (96). In cancer, overexpression of vimentin has been linked to tumor progression, metastasis, and unfavorable clinical outcomes (97). Interestingly, in eyelid sebaceous carcinoma, overexpression of vimentin triggers EMT, resulting in an adverse clinical prognosis. Furthermore, vimentin overexpression has emerged as a novel predictor of lymph node metastasis and poor survival (98).

### 5.4 Lymphoma

Distinct chromosomal anomalies have been observed in numerous lymphoproliferative and soft tissue tumors, as well as select benign orbital tumors. Identifying these specific chromosomal translocations is valuable for improving diagnosis and classification, particularly in cases with morphological similarities to other tumor types and low tumor differentiation. Gene dysregulation plays a crucial role in the pathogenesis of lymphoma. Johansson P (99) et al. have discovered several critical components of NF- κ B signaling that are frequently affected by genetic mutations in ocular appendage MALT type marginal zone lymphoma. Interestingly, over 60% of cases exhibited mutations in at least one NF- κ B regulator, while we also identified recurrent mutations in NOTCH1 and NOTCH2. These findings suggest that the abnormal NF- κ B signaling activation in ocular adnexal lymphoma may be attributed to recurrent chromosomal abnormalities, including the API2/MALT 1 and IG H/M ALT 1 translocation (100). These translocations ultimately result in the overexpression of MALT 1. Additionally, BCL10 positively regulates NF-κB signaling, while FOXP1 supports its activity. As a result, the NF-κB signaling pathway becomes hyperactivated and exhibits oncogenic activity (101).

Two primary subcategories of ocular adnexal lymphoma exist low-grade EMZL and aggressive diffuse large b-cell lymphoma (DLBCL). In exceptional cases, low-grade EMZL can transform into DLBCL. Hother C et al. discovered that dysregulation of miRNAs in the MYC and NF-κB pathways resulted in abnormal expression disparities between EMZL and DLBCL (102).

### 5.5 Melanoma

#### 5.5.1 DJ-1/PARK

Research has shown that DJ-1 acts as an oncogene and has transforming properties when combined with H-ras. This multifunctional protein protects cells from damage caused by reactive oxygen species and mitochondrial damage. Interestingly, there appears to be a correlation between DJ-1 levels and clinical risk factors for nevus growth. In UM, overexpression of the oncoprotein DJ-1/PARK 7 has been observed. Additionally, it has been discovered that the tumor can secrete soluble forms of this protein both *in vivo* and *in vitro*, which suggests that DJ-1 may serve as a potential biomarker for UM (103).

#### 5.5.2 KIF15

Kinesins, a type of motor protein, are often disrupted in various forms of cancer. Among the kinesin-12 family, KIF 15 is involved in several cellular functions such as differentiation, development, proliferation, and apoptosis. Recent research has discovered that KIF 15 is highly expressed in melanoma cells and tissues. Suppressing KIF 15 in tumors substantially reduced cell growth and increased apoptosis in both A375 and OCM 1 cell lines. Therefore, inhibiting KIF 15 can help diminish melanoma cell proliferation and promote apoptosis (104).

#### 5.5.3 PD-L1

Nuclear PD-L1 (nPD-L1) enhances EGR1-mediated angiogenesis by directly binding to the EGR1 promoter and upregulating its transcription, as demonstrated by RNA-seq and CUT&Tag data. This process is further amplified by phosphorylated STAT3 (p-STAT3), which acts as a coactivator with nPD-L1, leading to increased expression of EGR1 and its downstream target, VEGFA, both crucial for angiogenesis (105). The stabilization of PD-L1 by eEF2K, which prevents its degradation, strengthens PD-L1’s role in the tumor immune microenvironment and its correlation with better immunotherapeutic outcomes (106). The restoration of EGR1 in PD-L1-silenced cells rescues angiogenic and tumor growth capabilities, highlighting EGR1 as a key mediator in this pathway. These findings suggest that nPD-L1 drives a pro-angiogenic tumor environment through the EGR1/VEGFA axis, making it a potential target for therapeutic strategies (107).

Inhibition of the cystine/glutamate transporter xCT by sulfasalazine (SAS) or genetic knockdown induces ROS-related death in melanoma cells but simultaneously reduces the efficacy of anti-PD-1/PD-L1 therapy by upregulating PD-L1 expression via IRF4/EGR1 transcription factors, leading to exosome-mediated M2 macrophage polarization and subsequent resistance to the immune checkpoint blockade (108).

## 6 Development and variability of ocular surface and orbital tumors

Tumors form and progress due to changes and abnormalities in pathological tissues caused by various factors such as genetics, environment, and mutations. Pathology has evolved to include molecular biology, which has helped identify abnormal expression of genes, signaling pathways, and proteins contributing to tumor occurrence and development. However, relying solely on staining and fluorescence amplification of markers in tissue samples is not sufficient for independent tumor diagnosis. It is crucial to differentiate between benign and malignant tumors based on their biology, which can be determined through morphology and other indicators. Recent discoveries in molecular mechanisms have improved our understanding of tumor specificity, leading to better diagnoses and prognoses.

## 7 Conclusion

**Figure 1:**
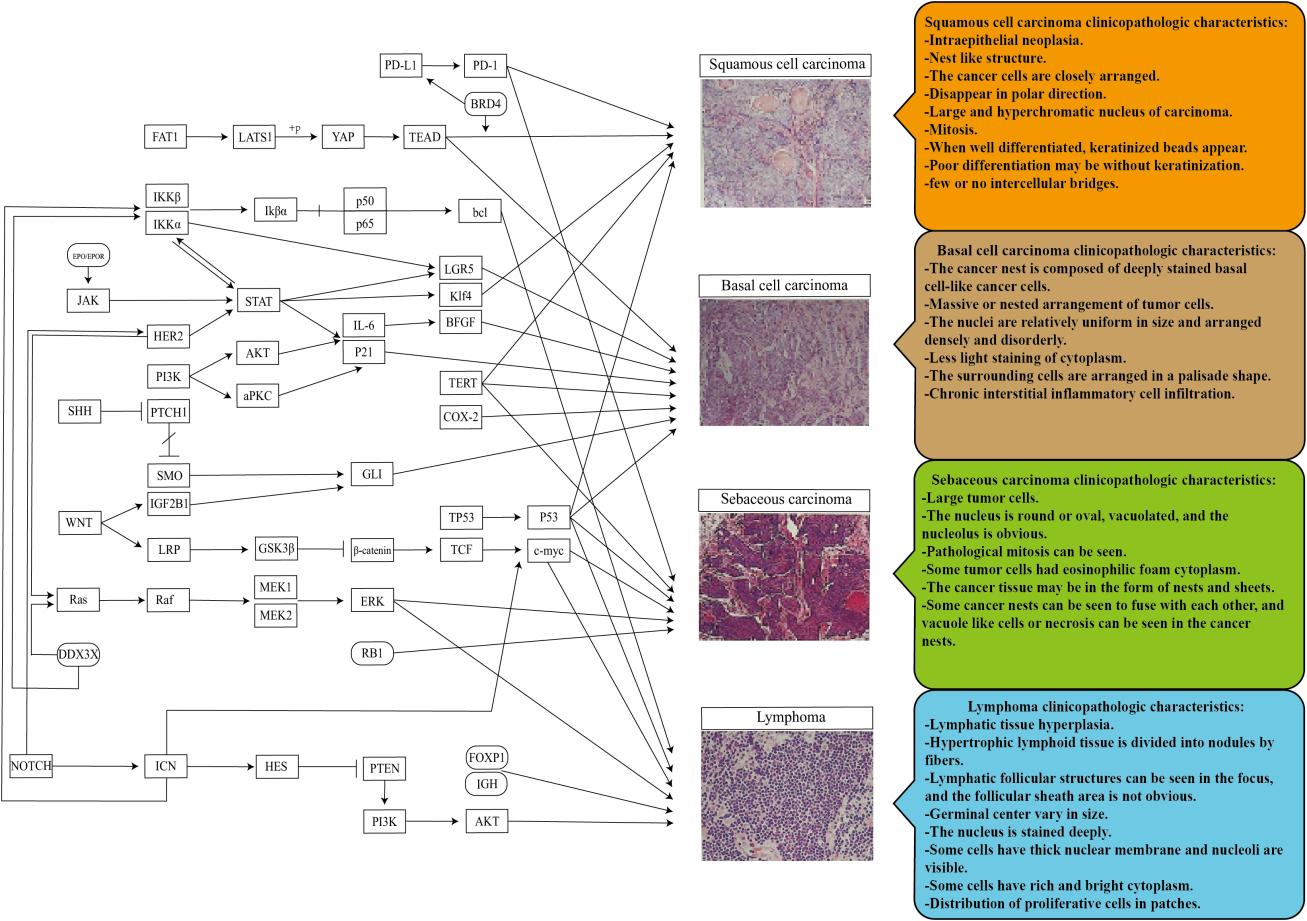
Pathogenic mechanism and pathological histological image characteristics of the ocular surface and orbital common malignant tumors. Promoting inhibition of different mechanisms in the tumor microenvironment enables the final transformation of normal tissues into different ocular tumors. There are similarities and differences in the pathological histology of different ocular tumors, a potential research direction for more tumor markers and translational medicine.

Ophthalmic pathology is a crucial component of the pathology system. Previous studies have revealed that eye tumors and tumors in other body parts share common or similar mechanisms. By examining clinical histopathology images, we can identify specific and shared microscopic characteristics of tumors (**Figure 1**). This suggests that we can identify independent diagnostic factors for tumor properties by collecting, classifying, and inducing data from clinical pathological histological images of tumors. In addition, epidemiological data and the molecular pathways are associated with the TME. Clinical workers can then use this information to assist in preliminary identifying unknown or difficult tumors as benign or malignant. This approach can facilitate precision medical care by informing subsequent specific marker checks (109). In addition, it is also hoped to provide a basis for thinking about and studying different mechanisms of future eye diseases.
